# Synthesis of DOTA-Based ^43^Sc Radiopharmaceuticals Using Cyclotron-Produced ^43^Sc as Exemplified by [^43^Sc]Sc-PSMA-617 for PSMA PET Imaging

**DOI:** 10.3390/mps8030058

**Published:** 2025-06-04

**Authors:** Jason P. Meier, Mohammed Bhuiyan, Richard Freifelder, Hannah J. Zhang, Lucas Gonzalez, Antonino Pusateri, Hsiu-Ming Tsai, Lara Leoni, Kaustab Ghosh, Erica Markiewicz, Christopher Henning, Yuhan Zhang, Ralph Weichselbaum, Jerry Nolen, David A. Rotsch, Chien-Min Kao, Russell Z. Szmulewitz, Chin-Tu Chen, Satish K. Chitneni

**Affiliations:** 1 Department of Radiology, The University of Chicago, Chicago, IL 60637, USA; mpbhuiyan@wisc.edu (M.B.); freifeld@uchicago.edu (R.F.); hjzhang@uchicago.edu (H.J.Z.); lcgonzalez@uchicago.edu (L.G.); npusateri@uchicago.edu (A.P.); kghosh@uchicago.edu (K.G.); ckao95@uchicago.edu (C.-M.K.); 2 Cyclotron Facility, The University of Chicago, Chicago, IL 60637, USA; 3 Department of Radiology, University of Wisconsin-Madison, Madison, WI 53792, USA; 4 UChicago/Argonne Joint Radioisotope Initiative (JRI), Chicago, IL 60637, USA; rweichselbaum@uchicagomedicine.org (R.W.); nolen@anl.gov (J.N.); rotschda@ornl.gov (D.A.R.); rszmulew@bsd.uchicago.edu (R.Z.S.); 5 Integrated Small Animal Imaging Research Resource, Office of Shared Research Facilities, The University of Chicago, Chicago, IL 60637, USA; tsai.hsiuming@gmail.com (H.-M.T.); lara.leoni@northwestern.edu (L.L.); ejmark@uchicago.edu (E.M.); 6 Center for Translational Imaging, Northwestern University, Chicago, IL 60611, USA; 7 Department of Medicine, The University of Chicago, Chicago, IL 60637, USA; christopher.henning@bsd.uchicago.edu (C.H.); yuhanz98@bsd.uchicago.edu (Y.Z.); 8 Department of Radiation and Cellular Oncology, The University of Chicago, Chicago, IL 60637, USA; 9 Physics Division, Argonne National Laboratory, Lemont, IL 60439, USA; 10 Medical Isotope Development Group, Oak Ridge National Laboratory, Oak Ridge, TN 37830, USA

**Keywords:** theranostics, Scandium-43, Scandium-44, Scandium-47, PSMA-617, prostate cancer, nuclear medicine, radiochemistry, oncology

## Abstract

The implementation of theranostics in oncologic nuclear medicine has exhibited immense potential in improving patient outcomes in prostate cancer with the implementation of [^68^Ga]Ga-PSMA-11 PET and [^177^Lu]Lu-PSMA-617 into clinical practice. However, the correlation between radiopharmaceutical biodistributions seen with [^68^Ga]Ga-PSMA-11 PET imaging and downstream [^177^Lu]Lu-PSMA-617 therapy remains imperfect. This suggests that prostate cancer theranostics could potentially be further refined through the implementation of true theranostics, tandem pairs of diagnostic and therapeutic radiopharmaceuticals that utilize the same ligand and element, thus yielding identical pharmacokinetics. The radioscandiums are one such group of true theranostic radiopharmaceuticals. The radioscandiums consist of two β+ emitting scandium isotopes (^43^Sc/^44^Sc), as well as a β^−^ emitting therapeutic isotope (^47^Sc), which can all conjugate with PSMA-targeting PSMA-617. This potential has led to extensive investigations into the production of the radioscandiums as well as pre-clinical assessments with several ligands; however, there is a lack of literature extensively describing the complete synthesis of scandium radiopharmaceuticals. which therefore limits the accessibility of radioscandium research in theranostics. As such, this work aims to present an easily translatable protocol for the synthesis of [^43^Sc]Sc-PSMA-617 from a [^42^Ca]CaCO_3_ starting material, including target formation, nuclear production via ^42^Ca(d,n)^43^Sc reaction, chemical separation, radiolabeling, solvent reformulation, and target recycling.

## 1. Introduction

The implementation of theranostics—synergistic pairs of radiopharmaceuticals consisting of a diagnostic and therapeutic element that exhibit highly similar pharmacokinetics—has been postulated to improve patient outcomes in oncologic nuclear medicine [1,2,3,4]. Because the diagnostic and therapeutic drugs in a theranostic pair have highly similar chemical structures and share the same biological target, the biodistribution of the diagnostic agent observed on Positron Emission Tomography (PET) or Single Photon Emission Computed Tomography (SPECT) imaging will have a direct correlation to the downstream biodistribution of the therapeutic agent. Consequently, theranostic imaging can predict the efficacy and dosimetric distribution of targeted radionuclide therapy (TRT) prior to the administration of any therapeutic drug, thus enabling better patient selection and more aggressive treatment planning [1,2,3,4].

Following the FDA approval and subsequent clinical usage of the prostate specific membrane antigen (PSMA) targeting [^68^Ga]Ga-PSMA-11 (LOCAMETZ) and [^177^Lu]Lu-PSMA-617 (PLUVICTO) for the staging and treatment of metastatic castration resistant prostate cancer (mCRPC), the potential of theranostics has most widely been observed in VISION substudies in which higher standardized uptake values (SUV) observed in lesions with [^68^Ga]Ga-PSMA-11 PET were moderately to strongly associated with improved outcomes with [^177^Lu]Lu-PSMA-617 therapy [5,6,7,8]. With regards to dosimetry, however, while exhibiting clear correlation, the ratio of observed dose determined by [^68^Ga]Ga-PSMA-11 PET and [^177^Lu]Lu-PSMA-617 SPECT has been shown to be variable in lesions as well as known organs at risk in [^177^Lu]Lu-PSMA-617 treatment, such as the kidneys and salivary glands [9,10]. One potential explanation for this inconsistency could be the differences in pharmacokinetics between [^68^Ga]Ga-PSMA-11 and [^177^Lu]Lu-PSMA-617 by virtue of their difference in both the chemical structure of the molecule and element. Therefore, by developing theranostic pairs with perfectly identical pharmacokinetics, known as true theranostics, it is possible to further refine the correlational accuracy of PET imaging and radionuclide therapy [1,2,3,4].

By nature of their identical pharmacokinetics and in vivo properties, the diagnostic and therapeutic components of a true theranostic pair must be conjugated to the same molecule and share the same element, varying only in radioisotope. The first implementation of true theranostics can therefore be attributed to the utilization of ^123^I and ^131^I for thyroid disease; however, the scope of true theranostics has since expanded to include the pre-clinical development of several receptor-targeted radiopharmaceuticals based on various true theranostic pairs such as ^64^Cu/^67^Cu, ^86^Y/^90^Y, ^203^Pb/^212^Pb, and the wide array of terbium radioisotopes (^149^Tb/^152^Tb/^155^Tb/^161^Tb) [11,12,13,14,15,16].

As scandium is a group-3 transition metal, much like ^177^Lu, the radioscandiums readily conjugate with the PSMA-targeting ligand, PSMA-617, thus making them prime candidates for true theranostic investigation, given the increasing interest in receptor-targeted true theranostics [17]. The radioscandiums are also composed of the necessary diagnostic and therapeutic components for theranostic applications, as they offer two β^+^ emitting radioisotopes capable of PET imaging in ^43^Sc and ^44^Sc as well as a β^−^ emitting therapeutic radioisotope that it is also capable of SPECT imaging in ^47^Sc [15,18,19,20]. ^43^Sc has a 3.891 h half-life and decays by β^+^ emission with an 88.1% frequency (the remaining 11.9% is attributed to electron capture) and an average β^+^ kinetic energy of 476.20 keV. ^43^Sc also emits a characteristic 372.9 keV γ-ray with 22.1% intensity, which facilitates identification with γ-ray spectroscopy [21]. Conversely, ^44^Sc has a comparable 4.042 h half-life, decays by β^+^ emission with 94.3% frequency, and emits a 1157.0 keV prompt γ-ray with 99.9% intensity [22]. This prompt γ-ray introduces additional considerations which must be made regarding PET detector saturation and dosimetry; therefore, we have elected to focus on ^43^Sc for the scope of this work [23,24]. As the therapeutic component of radioscandium theranostics, ^47^Sc decays by β^−^ emission with a mean β^−^ kinetic energy of 162.0 keV and 3.349 d half-life. ^47^Sc also emits a 159.4 keV γ-ray with 68.3% intensity, which can again facilitate isotopic identification with γ-ray spectroscopy [25].

There is extensive literature describing the production of the radioscandiums utilizing a multitude of proton, deuteron, gamma ray, and alpha particle production routes with both titanium and calcium targets, as well as preclinical evaluation of several scandium-based radiopharmaceuticals [18,26,27,28,29,30,31,32,33]. This includes isolated discussions of accelerator-based production and chemical separation methodology using calcium targetry, such as those described in Misiak et al. and Abel et al., as well as more longitudinal works which outline experimental results spanning from cyclotron production, to radiolabeling, and finally to the pre-clinical utilization of scandium radiopharmaceuticals in vitro and in vivo, such as those described in Van Der Meulen et al. and Domnanich et al. [34,35,36,37]. However, there has not yet been a study that provides a detailed procedure describing the complete synthesis of a scandium-based radiopharmaceutical from target material to ready-to-inject radiopharmaceutical in extensive and stepwise detail with the primary motivation being to facilitate the accessibility and reproducibility of radioscandium theranostic development. As such, this protocol aspires to fill that gap by describing the entirety of an optimized procedure to produce an injectable dose of [^43^Sc]Sc-PSMA-617 from a ^42^Ca starting material, including target preparation, radioisotope production (^42^Ca(d,n)^43^Sc), radiolabeling with PSMA-617, removal of any unreacted ^43^Sc, reformulation in PBS for preclinical experiments, and target material recovery. By providing a streamlined methodology for the synthesis of [^43^Sc]Sc-PSMA-617 from a calcium-based target, this protocol hopes to diminish the initial barriers to radioscandium research. As accessibility in research is critical for the widespread investigation of specific radiopharmaceuticals, it is therefore our hope that this protocol will further bolster investigation into the radioscandiums and thus the development of true theranostic radiopharmaceuticals as a whole [38].

## 2. Experimental Design

### 2.1. Protocol Overview

**Figure 1:**
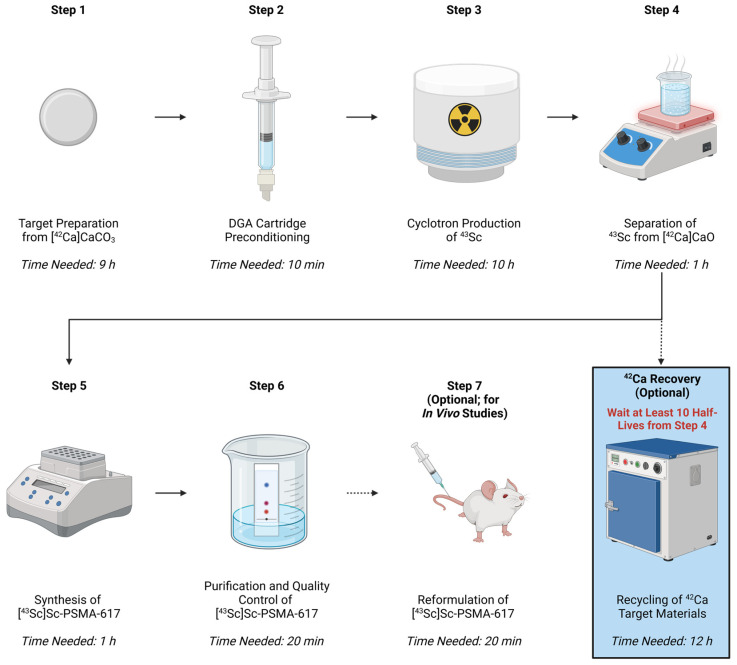
Graphical representation of the protocol to produce injectable [^43^Sc]Sc-PSMA-617 from [^42^Ca]CaCO_3_, as well as the subsequent protocol to recover the ^42^Ca starting materials from that synthesis. The singular step involving the downstream recovery of ^42^CaO from the waste products generated in Step 4 is highlighted in blue to signify its independence from the rest of the procedure. Created in BioRender by manuscript author Meier, J. (2025) https://BioRender.com/w18j428 (accessed on 30 April 2025).

This protocol describes a concise methodology for the synthesis of [^43^Sc]Sc-PSMA-617, which encompasses radioisotope production, radiolabeling, and the necessary downstream processing for the pre-clinical utilization of [^43^Sc]Sc-PSMA-617 in vitro and in vivo. The protocol is subsequently broken down into the following sequential steps: target preparation from [^42^Ca]CaO_3_; cyclotron production of ^43^Sc; DGA cartridge preparation; separation of ^43^Sc from [^42^Ca]CaO; synthesis of [^43^Sc]Sc-PSMA-617; purification and quality control of [^43^Sc]Sc-PSMA-617; and reformulation of [^43^Sc]Sc-PSMA-617. An additional protocol for the post-production recovery of [^42^Ca]CaO target material adopted from US Patent 20170087260 (Paul Scherrer Institute, Würenlingen, Switzerland) application is also presented [39]. The synergistic utilization of this target recovery protocol is highly recommended to minimize the material cost of [^43^Sc]Sc-PSMA-617 production, given the scarcity of isotopically enriched ^42^Ca. The workflow and estimated timing of both the [^43^Sc]Sc-PSMA-617 and [^42^Ca]CaO recovery procedures are presented in Figure 1.

### 2.2. Translation to Other Scandium Isotopes and DOTA-Based Radioligands

This protocol describes methodology for the synthesis of ^43^Sc and the subsequent radiosynthesis of [^43^Sc]Sc-PSMA-617; however, the procedures outlined in this manuscript are also applicable to the synthesis of other scandium-based radiopharmaceuticals. Notably, steps 1–9 and 13–40 described in “Production of [^43^Sc]Sc-PSMA-617 using cyclotron produced ^43^Sc” are directly transferable to the radiosynthesis of Sc-PSMA-617 with other scandium radioisotopes that can be produced from a CaO target. Our group has utilized this same procedure to produce [^44^Sc]Sc-PSMA-617 with the ^44^Ca(p,n)^44^Sc reaction and [^47^Sc]Sc-PSMA-617 through the ^48^Ca(p,2n)^47^Sc reaction. These expanded applications of this procedure do not require any procedural modifications aside from the utilization of a proton beam rather than deuteron beam during the cyclotron irradiation. In accordance with the change in beam particle, the application of this protocol to the production of ^44^Sc or ^47^Sc will also require the use of different incident beam energies than is appropriate for ^43^Sc production. Notably, the ^44^Ca(p,n)^44^Sc cross-section is maximized with a proton beam energy between 7–14 MeV; however, it may be desirable to utilize a beam energy below 13 MeV to minimize the pr/oduction of metastable ^44m^Sc [40,41,42]. Conversely, the ^48^Ca(p,2n)^47^Sc cross-section peaks between a proton beam energy of 14–20 MeV [41,42].

As a means of obtaining familiarity with the chemical procedure described in this work prior to introducing the additional challenges and hazards brought forth by the handling of radioactive materials, it is also possible to emulate the chemical separation, radiolabeling, purification, and reformulation portions of this protocol by artificially adding stable natural scandium metal powder to natural CaO and following the steps described in Section 3.1.4, Section 3.1.5, Section 3.1.6 and Section 3.1.7. However, as the validation methods presented throughout this work utilize the radioscandium decay radiation as a proxy for the identification of the desired products, new validation methods will have to be designed to replace the use of well counter activity measurements, radio-TLC, and radio-HPLC.

Additionally, the radiolabeling procedure described in steps 26–41 can be more broadly applied to the synthesis of other scandium radiopharmaceuticals that use DOTA as the chelation site, so long as the chosen ligand can withstand the temperatures and pH necessary to facilitate the binding of scandium to the chelation complex [43,44,45,46,47]. Recent investigation into [^43^Sc]Sc-DOTATATE and [^47^Sc]Sc-DOTATATE are prime examples of this translatability and are of particular interest due to being direct analogues of [^68^Ga]Ga-DOTATATE and [^177^Lu]Lu-DOTATATE [24,48,49].

### 2.3. Materials

- Isotopically enriched [^42^Ca]CaCO_3_ (Isoflex USA, San Francisco, CA, USA)
- Trance analysis water (Honeywell, Charlotte, NC, USA; P/N 95305500ML or equivalent)
- HCl (trace metal grade) (Fisher Chemical, Pittsburgh, PA, USA; P/N A508-P500 or equivalent)
- HNO_3_ (trace metal grade) (Fisher Chemical, Pittsburgh, PA, USA; P/N A509P212 or equivalent)
- Acetic Acid (trace metal grade) (Fisher Chemical, Pittsburgh, PA, USA; P/N A507-P500 or equivalent)
- Ammonium Acetate (trace metal grade) (Sigma Aldrich, St. Louis, MO, USA; P/N 50-180-4364 or equivalent)
- Ammonium Formate (HPLC grade) (Honeywell, Charlotte, NC, USA; P/N 1784350G or equivalent)
- Methanol (HPLC Grade) (MilliporeSigma, Burlington, MA, USA; P/N MMX0475P6 or equivalent)
- Ethanol (200 proof)
- PSMA-617 (Vipivotide tetraxetan) (Selleckchem, Houston, TX, USA; P/N S8670)
- Phosphate-buffered saline (PBS)
- Ammonium oxalate monohydrate (trace-analysis grade) (Thermo Scientific Chemicals, Waltham, MA, USA; P/N 206275000 or equivalent)
- Ammonium Hydroxide (trace metal grade) (Fisher Chemical, Pittsburgh, PA, USA; P/N A512-P500 or equivalent)
- Non-Branched DGA Resin 2 mL Column (Eichrom, Lisle, IL, USA; P/N DN-R50-S)
- Oasis HLB light cartridge (30 mg sorbent) (Waters, Milford, MA, USA; P/N 186005125)

### 2.4. Equipment

- PTFE Coated Spatula (Fisher Scientific, Pittsburgh, PA, USA; P/N 13-820-058 or equivalent)
- Alumina crucible or combustion boat and cover (Fisher Scientific, Pittsburgh, PA, USA; P/N FB960I or equivalent)
- Box furnace (Lindberg/MPH, Riverside, MI, USA; BF51800 or equivalent)
- Pellet press and corresponding punch and die set (Parr Instrument Company, Moline, IL, USA; P/N 2810-2106-103101)
- Accumet AB15+ pH/mV/°C meter (Fisher Scientific, Pittsburgh, PA, USA; P/N 13-636-AB15PB or equivalent)
- Isotemp thermal mixer (Fisher Scientific, Pittsburgh, PA, USA; P/N 270600F or equivalent)
- Scan-Ram radioTLC scanner (LabLogic, Sheffield, UK)
- iTLC SG glass microfiber chromatography paper (Agilent Technologies, Santa Clara, CA, USA; P/N SGI0001)
- Infinity 1260 HPLC system (Agilent Technologies, Santa Clara, CA, USA; P/N 9572)
- Flow-Ram radioHPLC detector (LabLogic, Sheffield, UK)
- Wizard2 2480 gamma counter (Revvity, Waltham, MA, USA)
- Magnetic stirrer hot plate
- Microcentrifuge (compatible with 2 mL tubes)
- Fume hood with a nitrogen gas line
- Buchner funnel with inner joint
- Buchner funnel with filter disc
- 1000, 100, and 10 µL micropipettes

### 2.5. Laboratory Supplies

- Antistatic polystyrene weighing dishes (Fisher Scientific, Pittsburgh, PA, USA; P/N 08-732-112 or equivalent)
- 25 mL PFA beaker (Corning, Corning, NY, USA; P/N 1003P-25 or equivalent)
- 15 mL conical centrifuge tubes (Corning, Corning, NY, USA; P/N 352097 or equivalent)
- 2 mL microcentrifuge tubes (Fisher Scientific, Pittsburgh, PA, USA; P/N 05-408-138 or equivalent)
- Screw top septum vials (Fisher Scientific, Pittsburgh, PA, USA; P/N PI13019 or equivalent)
- Polystyrene tubes for Wizard2 2480 (Revvity, Waltham, MA, USA; P/N 50-905-2501)
- Whatman 42 quantitative ashless filter paper, GR42 (Cytiva, Marlborough, MA, USA; P/N 1442042)
- 1000, 100, and 10 µL filtered pipette tips
- Kimwipes
- Parafilm

## 3. Procedure

### 3.1. Synthesis of Injectable [^43^Sc]Sc-PSMA-617 from [^42^Ca]CaCO_3_—Total Time Needed: 22 h

#### 3.1.1. Target Preparation from [^42^Ca]CaCO_3_—Time Needed: 9 h


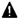
 **CRITICAL STEP** The transfer of solid reagents described in this section (along with any transfers in subsequent sections) should only be performed with non-metal or PTFE coated tools. Failure to use non-metal or coated tools may result in metal contamination in the ^43^Sc solution. Metal contaminants will compete with ^43^Sc for the chelation complex in the radiolabeling portion of this procedure and may result in failed conjugation with PSMA-617 [46,48].

**Figure 2:**
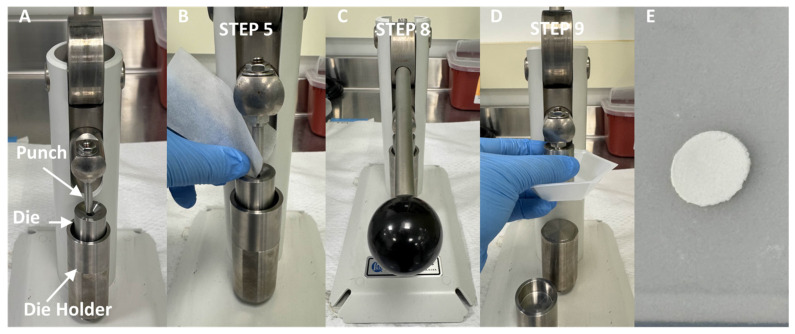
A pictorial representation of the major steps of target formation that utilize the target press. (**A**) The major parts of the pellet press. (**B**) Loading the pellet press with [42Ca]CaO powder using a weighing paper, as is described in Step 5. (**C**) Pressing the target with the punch, as described in Step 8. (**D**) Retrieval of the [42Ca]CaO target, as described in Step 9. (**E**) The final [42Ca]CaO produced at the conclusion of Step 9.

1. Transfer [^42^Ca]CaCO_3_ to an alumina crucible or combustion boat.
  - When deciding the amount of [^42^Ca]CaCO_3_ to transfer, consider that calcination removes CO_2_ to produce [^42^Ca]CaO and thus the final target mass will be significantly lower than the mass of the starting material (1.0 mg of CaCO_3_ yields 0.56 mg of CaO).
  - A quartz crucible can also be utilized in place of an alumina crucible; however, a quartz crucible will incur a significant buildup of static electricity, making it difficult to transfer the resultant [^42^Ca]CaO without loss.
2. Place the [^42^Ca]CaCO_3_ in a laboratory furnace and gradually increase the furnace temperature to 800 °C in 100–150 °C increments.
3. Maintain a temperature of 800 °C for 1 h to ensure complete conversion of [^42^Ca]CaCO_3_ to [^42^Ca]CaO.
4. Shut off the furnace and allow it to return to room temperature (~ 8 h) before removing the crucible containing [^42^Ca]CaO.
5. Scrape the [^42^Ca]CaO material from the crucible onto a piece of weighing paper. Carefully use the weighing paper to transfer the material into the die of the pellet press, as is shown in Figure 2.

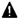
 **CRITICAL STEP** Ensure that the pellet press is free of residual material prior to use by wiping the die, die holder, and punch of the pellet press with an ethanol soaked Kimwipe. The presence of residual calcium on any of these parts can cause the resultant target to become brittle or malformed due to an uneven distribution of pressure during pressing

6. Adjust the height of the die such that a non-negligible amount of force is required to fully press the punch into the bottom of the die.

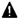
 **CRITICAL STEP** Improperly adjusting the die height will lead to broken targets (die is too high and thus too much pressure is exerted by the punch) and malformed targets (die is too low and not enough pressure is exerted by the punch).

7. Lower the punch into the die until the point that external force beyond gravity would be required to lower it any further. Gently rotate the die around the punch to evenly distribute the [^42^Ca]CaO material in the die.
8. Fully press the punch down into the die.
9. Lift the punch out of the die and retrieve the newly formed target.

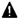
 **CRITICAL STEP** As the desired thickness of the target decreases, the structural integrity of the target decreases, making it substantially more difficult to maintain the desired disc shape of the target (which maximizes the target surface area in contact with the deuteron beam and thus maximizes yield). Therefore, if attempts to press a target are resulting in broken or imperfect discs, it is recommended to increase the mass of target material being pressed to reduce brittleness.

It is also common for the target to become stuck in the die after pressing. If this occurs, carefully remove the die from the die holder and hold a weighing dish underneath the die while gently running it through the punch.

If the target comes out of the die into the die holder during pressing (as intended), it is recommended to remove the target by placing a weighing dish over the die holder and inverting it. Attempting to remove the target with a spatula or forceps will likely result in fragmentation of the target.

10.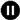 **PAUSE STEP** The calcified [^42^Ca]CaO target can be stored indefinitely in a parafilm wrapped weighing dish if it is kept under vacuum. This prevents the reabsorption of atmospheric carbon dioxide (reforming CaCO_3_) or water (forming Ca(OH)_2_) into the target. If the target has been exposed to atmospheric air for a prolonged period, the target can be re-calcinated into CaO by repeating the above procedure.

#### 3.1.2. DGA Cartridge Preconditioning—Time Needed: 10 min


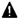
 **CRITICAL STEP** Work described in this section should only be performed with metal-free or trace-analysis grade reagents. Failure to use such reagents may result in metal contamination in the ^43^Sc solution. Metal contaminants will compete with ^43^Sc for the chelation complex in the radiolabeling portion of this procedure and may result in failed conjugation with PSMA-617 [46,48].


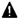
 **CRITICAL STEP** The preconditioning of the DGA cartridge described in this section is a procedure which must be executed at time-sensitive points relative to other portions of this protocol, notably the separation of ^43^Sc from the ^42^Ca target which begins in Section 3.1.4. Because it requires a slow gravity drip, the first phase of conditioning (Step 11) must be initiated approximately 24 h prior to the start of the separation of ^43^Sc from the ^42^Ca target. Failure to allow the DGA cartridge to gravity drip for enough time can lead to the cartridge being improperly conditioned, ultimately resulting in a failed trapping or elution of the desired [^43^Sc]ScCl_3_ product in Section 3.1.4.


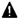
 **CRITICAL STEP** The passing of any solution through the DGA column in Section 3.1.2. and Section 3.1.4. is done by loading the cartridge from the top and pushing the solution out from the bottom of the cartridge. Use of the reverse orientation (loading from the bottom and eluting from the top) may result in the unsuccessful trapping or elution of the ^43^Sc product.

11. Draw 6 mL (three full-column volumes) of 2.5 M HNO_3_ into a needleless syringe. Connect the top of the DGA cartridge to the syringe and gently saturate the sorbent with HNO_3_. Vertically place the cartridge inside a conical tube so it can gravity drip for approximately 24 h.
12. Slowly push the remaining 2.5 M HNO_3_ through the cartridge at an approximate rate of 1.0 mL/s. Fill another needleless syringe with 6 mL (three ful- column volumes) of 5 M HNO_3_ and use it to gently wash the DGA column.

#### 3.1.3. Cyclotron Production of ^43^Sc—Time Needed: 10 h

13. Transfer the [^42^Ca]CaO target into the cyclotron’s solid target holder and load it into the cyclotron.
  - If using a retrofitted target holder, such as the beam stop-based target holder we describe in Meier et al., it is helpful to wrap the target in a thin graphite foil prior to transfer [50]. This does not impact any of the downstream separation chemistry and ensures that no target material is lost during loading or retrieval [50].
14. Produce ^43^Sc via the ^42^Ca(d,n)^43^Sc reaction by irradiating the target with a deuteron beam. The deuteron current, irradiation time, and incident deuteron energy can all be adjusted based upon the desired activity amount and the cooling capabilities of the solid target holder. Increasing beam current and irradiation time will result in larger yields. The TENDL simulated ^42^Ca(d,n)^43^Sc cross-section suggests that the most optimal yields occur between 2–10 MeV [42]. For this publication, we have utilized a beam energy of 9 MeV, 2–10 μA of beam current, and an irradiation time of 8–10 h.
15. Approximately 1 h after the end of bombardment, retrieve the irradiated target from the cyclotron.

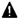
 **CRITICAL STEP** It is essential that the target is kept inside the shielded cyclotron vault immediately after irradiation, so that the short-lived isotopes produced can decay prior to handling. While the abundance of these short-lived isotopes will vary with the isotopic composition of the target material and selected beam energy, potential short-lived contaminants originating from the target include: ^46^K (t_1/2_ = 96.31 s), ^49^Ca (t_1/2_ = 8.718 min), ^44^K (t_1/2_ = 22.13 min), and ^49^Sc (t_1/2_ = 57.20 min) [41,42,50]. If using a solid target holder retrofitted to the beam stop, such as that described in Meier et al., any titanium components in the target holder would also result in the production of short-lived ^47^V (32.6 min) [41,42,50]. Although not well characterized, as the production of ^43^Sc involves the ejection of a neutron from ^42^Ca, neutron activation of the cyclotron vault and aluminum in the target holder is also possible. With regards to the target holder this would result in the production of ^28^Al (t_1/2_ = 2.25 min) and ^29^Al (t_1/2_ = 6.56 min) [51,52]. Ignoring the critical 1 h buffer time will lead to a significant, unnecessary dose to any personnel handling the target.

16. Ensure the quantity and identity of the resultant ^43^Sc by measuring the activity in a well counter and confirming the identity of the radioactive source with high purity germanium (HPGe) detector or cadmium zinc telluride (CZT) gamma spectroscopy. Peaks should be observed for the characteristic 372.9 keV gamma emission as well as the 511 keV positron annihilation [41].

#### 3.1.4. Separation of ^43^Sc from [^42^Ca]CaO—Time Needed: 1 h


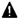
 **CRITICAL STEP** Work described in this section should be performed in a shielded fume hood or hot cell to protect staff from radioactive and chemical hazards.


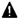
 **CRITICAL STEP** Work described in this section should only be performed with metal-free or trace-analysis grade reagents. Failure to use such reagents may result in metal contamination in the ^43^Sc solution. Metal contaminants will compete with ^43^Sc for the chelation complex in the radiolabeling portion of this procedure and may result in failed conjugation with PSMA-617 [46,48].


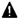
 **CRITICAL STEP** As this portion of the procedure involves the use of highly concentrated acids, pipette tips with filters should be utilized to minimize the risk of damage to pipettes from acid fumes.


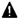
 **CRITICAL STEP** The passing of any solution through the DGA column in Section 3.1.2. and Section 3.1.4. is done by loading the cartridge from the top and pushing the solution out from the bottom of the cartridge. Using the reverse orientation (loading from the bottom and eluting from the top) may result in the unsuccessful trapping or elution of the ^43^Sc product.

17. Fill a conical tube with 10 mL of 0.1 M HCl and place it in a water bath heated to 95 °C. Once the 0.1 M HCl also reaches a temperature of 95 °C, it will be used in Step 24.

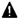
 **CRITICAL STEP** While the water used in this step is not involved in any chemical reactions, it is preferable to utilize MilliQ or trace-analysis grade water to minimize metal contamination should any droplets come into contact with the HCl.

18. Transfer the irradiated [^42^Ca]CaO/^43^Sc target to a 25 mL PFA beaker. Add 2 mL of 15 M HNO_3_ to the beaker containing the target and place the resulting solution on a hotplate preheated to 70 °C.

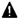
 **CRITICAL STEP** Carefully monitor the temperature throughout this dissolution process. If the HNO_3_ begins to boil, there is a significant risk that some activity will be carried away with the vapor, resulting in a contaminated laboratory space.

Additionally, it is crucial that the hotplate is preheated to the target temperature prior to placing the target solution on it. If the hotplate is instead heated while the target solution is in contact with it, temperature overshoot can occur, causing the solution to boil.

19. Once the [^42^Ca]CaO/^43^Sc solution is on the hot plate, add 1 mL of water every 8 min until 4 mL of water has been added, bringing the final dissolution volume to 6 mL and HNO_3_ concentration to 5 M. Allow the solution to remain under heat for an additional 8 min.
20. Remove the [^42^Ca]CaO/^43^Sc solution from the hotplate. Collect the solution with a micropipette, taking care to avoid any pieces of graphite foil that broke off during dissolution. Slowly load the solution into the DGA cartridge at a rate of approximately 0.2 mL/s. Collect the pass-through solution in a conical tube. As the branched DGA extractant (N,N,N’,N’-tetrakis-2-ethylhexyl-diglycolamide) has a high affinity for trivalent metals under acidic conditions, the ^43^Sc in the highly acidic dissolution solution should bind tightly to the DGA resin while the ^42^Ca target material passes through. Preserve the PFA beaker and its remaining contents in a shielded area, as they will be used in the target recovery process.
21. Assay the pass-through solution with a well counter to ensure that the radioactive ^43^Sc has successfully been trapped by the DGA cartridge. Place the conical tube containing the pass-through solution in a shielded area so any trace radioactivity can decay out and the solution can be retained for ^42^Ca recovery.

If a large fraction of the overall activity was detected in the pass-through solution (>30%), attempt to re-load the collected pass-through solution into the DGA cartridge as slowly as possible without incurring a significant radiation dose. Re-assay the new resulting pass-through solution. If this does not result in the sufficient trapping of ^43^Sc, it is likely that the DGA cartridge was not conditioned correctly, and thus the activity in the pass-through solution is irrecoverable within the scope of the ^43^Sc half-life.

22. Gently wash the DGA column with 6 mL of 5 M HNO_3_. Collect and assay the washings to ensure that the ^43^Sc was not prematurely eluted. Again, preserve the washings in a shielded area, as they will be used in the target recovery process.
23. Gently wash the DGA column with 6 mL of 1 M HCl. Collect and assay the washings to ensure that the ^43^Sc was not prematurely eluted. Again, preserve the washings in a shielded area, as they will be used in the target recovery process.
24. Fill a needleless syringe with 5 mL of the 95 °C 0.1 M HCl from Step 17. Attach the DGA column to the syringe and gently elute out the [^43^Sc]ScCl_3_ product into approximately ten 500 µL aliquots, each in their own 2 mL centrifuge tube. As the introduction of a weak acid mobile phase to the DGA column greatly reduces its affinity for trivalent cations, this should result in the majority of the ^43^Sc atoms dissociating from the extractant, binding to the free chloride ions, and being eluted out from the column in the weak acid solution; however, a small amount of ^43^Sc retention in the column (<10%) is expected.

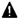
 **CRITICAL STEP** Highly concentrated fractions of radioactivity are critical for successful radiolabeling with high molar activity, as is mandated by meaningful in vitro and in vivo experiments. As such, it is important that the elution of [^43^Sc]ScCl_3_ is performed slowly (approximately 0.1 mL/s), such that most of the activity is eluted in the first few aliquots and minimal activity is remnant in the column.


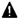
 **CRITICAL STEP** It is easy to contaminate the workspace when moving the syringe and column from one aliquot to the next. To minimize contamination, release any pressure you are placing on the plunger and move the syringe between aliquots only when it is between drops of the eluent being released from the syringe.

25. Assay each aliquot in the dose calibrator to determine which have the highest concentration of radioactivity. Assay the DGA column to ensure a significant amount of activity has not remained in the column. Preserve the DGA column in a shielded area, as it will be used in the target recovery process.
26. If a large fraction of radioactivity has been retained in the DGA column (>10%), it may be necessary to elute additional fractions with 95 °C 0.1 M HCl until enough activity has been obtained for any downstream experiments. However, it is likely that these fractions will be of a lower activity concentration than those previously obtained and, therefore, suboptimal for radiolabeling.

#### 3.1.5. Synthesis of [^43^Sc]Sc-PSMA-617—Time Needed: 1 h


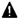
 **CRITICAL STEP** Work described in this section should be performed in a shielded fume hood or hot cell to protect staff from radioactive and chemical hazards.


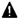
 **CRITICAL STEP** Work described in this section should only be performed with metal-free or trace-analysis grade reagents. Failure to use such reagents may result in metal contamination in the ^43^Sc solution. Metal contaminants will compete with ^43^Sc for the chelation complex in the radiolabeling portion of this procedure and may result in failed conjugation with PSMA-617 [46,48].

27. Preheat the thermal mixer to 95 °C.
28. Add an equal volume of 1 M ammonium acetate buffer (pH 5.25) to the [^43^Sc]ScCl_3_ aliquots that will be used for labeling the PSMA-617 ligand. Use a disposable pH paper to check that the pH of the buffer-activity solution is between pH 4–5, as that is the range where the conjugation of ^43^Sc to the DOTA chelator is most favorable. If the pH is too low, add small amounts of 0.5 M ammonium acetate buffer (pH 5.25) until the pH reaches 4.

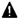
 **CRITICAL STEP** When using pH paper to check the pH of the buffer-activity solution, it is best to cut the paper into thin strips and then wet them with 2–5 µL using a micropipette. This ensures that contaminants are not introduced into the reaction mixture by the pH paper, and minimizes activity loss.

29. Utilizing the activity data collected in Step 25, calculate the amount of PSMA-617 ligand that must be added to the buffer-activity solution to achieve the desired molar activity, according to Equation (1) below. Add the calculated amount of ligand (dissolved in water; see reagent preparation) to the buffer-activity solution.
  (1) $${Volume}_{Ligand}(µL)=\frac{Activity\;\left(MBq\right)\times Molecular\;Weight\;\left(\frac{ng}{nmol}\right)}{Molar\;Activity\;\left(\frac{MBq}{nmol}\right)\times Density_{Aqueous\;Ligand}\left(\frac{µg}{µL}\right)}\times \frac{1\;(µg)}{1000\;(ng)}$$

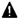
 **CRITICAL STEP** The maximum achievable molar activity will be heavily contingent upon the activity concentration within the reaction mixture as a consequence of Le Chatelier’s principle [53]. The amount of metal contaminants within the reaction mixture will also greatly influence the maximum achievable molar activity due to the high affinity of PSMA-617’s DOTA chelation complex for heavy metals such as iron, copper, and zinc [54]. As such, one should consider these two factors when selecting a target molar activity, and, even more so, as potential explanations if a radiolabeling reaction fails at a given target molar activity. Consequently, we recommend users of this protocol begin their implementation of this protocol with a low target molar activity (for example, 1 MBq/nmol), and then incrementally increase the target molar activity with each sequential experiment.

30. Place the reaction mixture into the preheated thermal mixer. Leave the temperature at 95 °C and set the mixing frequency to ~500 RPM. Allow the reaction to occur under these conditions for 40 min.
31. Once the reaction time has elapsed, check that [^43^Sc]Sc-PSMA-617 has been formed by spotting on an iTLC-SG paper with approximately 2 µL of the reaction mixture. Develop the spotted iTLC paper in a mobile phase consisting of a 1:1 ratio of methanol and 1 M ammonium formate. Once developed, analyze the iTLC plate with a radioTLC scanner. Unlabeled ^43^Sc should remain near the spotting line, whereas [^43^Sc]Sc-PSMA-617 will migrate with the solvent front.

#### 3.1.6. Purification and Quality Control of [^43^Sc]Sc-PSMA-617—Time Needed: 20 min


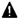
 **CRITICAL STEP** Work described in this section should be performed in a shielded fume hood or hot cell to protect staff from radioactive and chemical hazards.

32. Condition a Waters Oasis HLB light cartridge (30 mg sorbent) by slowly pushing 3 mL of ethanol followed by 6 mL of water at a rate of approximately 1.0 mL/s.
33. Load the radiolabeled solution into the conditioned Oasis cartridge and slowly pass it through. Collect and assay the pass-through in a well counter to ensure that the [^43^Sc]Sc-PSMA-617 has been retained on the cartridge. Any unlabeled ^43^Sc should be eluted in the pass-through solution and subsequent wash described in Step 33.
34. Wash the Oasis cartridge with 2 mL of water. Collect and assay the washings to ensure that the [^43^Sc]Sc-PSMA-617 has not prematurely eluted.
35. Slowly elute the [^43^Sc]Sc-PSMA-617 solution with 300 µL of ethanol at an approximate rate of 0.05 mL/s. If the activity will be utilized for an animal injection, it is recommended to elute into a screw top septum vial (e.g., 4 mL) as is necessary for steps 37–41.
36. Repeat Step 30 with the now-purified [^43^Sc]Sc-PSMA-617 solution to ensure that all unlabeled ^43^Sc has been removed. The subsequent iTLC should now have only a single peak that moves with the solvent front.
37. If reformulation of the [^43^Sc]Sc-PSMA-617 is unnecessary, such as in cellular studies where the ethanol will be greatly diluted, transfer 25 µL of the final purified solution into a small V-vial. From this V-vial, inject 5 µL into the HPLC system for radio-HPLC analysis. The resultant chromatogram can be compared to a standard curve generated by the UV trace of a reference solution of cold PSMA-617 to calculate the true molar activity of the final solution.

#### 3.1.7. Reformulation of [^43^Sc]Sc-PSMA-617—Time Needed: 20 min


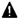
 **CRITICAL STEP** Work described in this section should be performed in a shielded fume hood or hot cell to protect staff from radioactive and chemical hazards.

**OPTIONAL STEP** This section pertains to the reformulation of the [^43^Sc]Sc-PSMA-617 solvent so that it is acceptable for utilization in in vivo studies. Only proceed with this procedure if necessary for downstream experiments.

38. Preheat a hot plate to 60 °C.

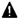
 **CRITICAL STEP** It is critical that the hotplate is preheated to the target temperature prior to placing the ethanol solution on it. If the hotplate is instead heated while the target solution is in contact with it, temperature overshoot can occur, causing the solution to boil too rapidly, thus yielding a buildup of excessive pressure within the vial and increasing the surface area the radioactive solution comes into contact with (reducing downstream yield).

39. Transfer the ethanol solution containing [^43^Sc]Sc-PSMA-617 produced in Step 34 to a screw top septum vial if it is not already in one. Place the vial on the preheated hotplate. Attach a 20-gauge needle to a nitrogen line and carefully insert it into the septum, ensuring that the needle is not touching the radioactive solution. Insert another 18-gauge needle into the septum for venting. Turn on a gentle flow of nitrogen and evaporate the ethanol medium to near dryness.
40. Use a needle and syringe to add the desired reconstituting medium to the vial at the desired activity concentration. For PET imaging, we recommend using a ratio of approximately 0.5 µL of PBS for every 0.037 MBq of radioactivity.
41. Utilize a disposable pH paper to check that the pH of the final dose is near the blood pH of the experimental group (pH ~7–7.4 in mice). If the measured pH falls outside the physiological range, continue to add PBS until the pH becomes a safe level for in vivo use.

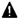
 **CRITICAL STEP** As the final reformulated dose is intended for in vivo use, it is critical that the pH is checked before in vivo application after every synthesis. Injection of a radiopharmaceutical in a non-physiological solvent could cause suffering, physical harm, and death in the experimental subject [55,56]. This is especially true when conducting in vivo studies with animals that have a low blood volume, such as mice, or animals with impaired renal function, as is the case in animals with tumor xenografts [56].


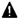
 **CRITICAL STEP** When using pH paper to check the pH of the buffer-activity solution, it is best to cut the paper into thin strips and then wet them with 2–5 µL using a micropipette. This ensures that contaminants are not introduced into the reaction mixture by the pH paper, and minimizes activity loss.

42. Analyze an aliquot of the reconstituted solution by radio-HPLC as described in Step 36.

### 3.2. Recycling of ^42^Ca Target Materials—Time Needed: 12 h


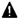
 **CRITICAL STEP** Prior to beginning the recovery procedure, assay the recovery materials collected in Steps 20–25 and ensure that they are no longer radioactive and thus safe for handling. Performing target recovery with radioactive materials will lead to an unnecessary dose to staff and potential contamination of the laboratory space.


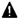
 **CRITICAL STEP** It is recommended to combine and recover the ^42^Ca from multiple productions simultaneously rather than performing a recovery after each individual production. This is because each transfer of the ^42^Ca recovery materials that is performed will result in the loss of some ^42^Ca. By minimizing the number of times the procedure is performed, the number of transfers is minimized as well.

1. Wash the used DGA column from Step 25 in Section 3.1.4. with 10 mL of 3 M HCl. Collect the washings in an approximately 200 mL beaker.
2. Combine the DGA pass-through, 1 M HCl wash, and 5 M HNO_3_ wash from Steps 21–23 of Section 3.1.4. in the 200 mL beaker. Also wash each of the conical tubes that contained these solutions with 2 mL of metal-free water and collect those washings in the 200 mL beaker.
3. Wash the PFA beaker that was used in Step 20 of Section 3.1.4. with 2 mL of metal-free water and collect the washings in the 200 mL beaker.
4. **OPTIONAL STEP** Store any used recovery materials from the previous four steps such that they are available if a secondary recovery is necessary.
5. Add a large stir bar to the 200 mL beaker and place it on a hot plate set to approximately 150 °C with gentle stirring. Carefully evaporate the solution to dryness.

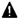
 **CRITICAL STEP** It is important to evaporate the solution slowly, as vigorous boiling can cause portions of the recovery solution to splash outside of the beaker, resulting in a loss of ^42^Ca material.


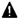
 **CRITICAL STEP** It is critical to quickly remove the dried solution from the hot plate as soon as it reaches complete dryness. Should the dry solution be left on the hot plate too long, it can undergo incomplete combustion, yielding a charred substance. This charring will inhibit the downstream recovery processes.

6. Redissolve the dried solution in 5 mL of 3 M HCl and repeat the previous step (Step 5) of this procedure.
7. Once fully dried, add 20 mL of water to the 200 mL beaker to redissolve the calcium.
8. Adjust the pH of the calcium solution to pH 4.5–5 using 2.5% ammonium hydroxide and 1 M HCl.
9. Add 20 mL of 0.3 M ammonium oxalate to the pH adjusted solution, yielding calcium oxalate. Upon adding the ammonium oxalate, the solution should turn an opaque, cloudy white color.
10. Allow the calcium oxalate solution to stand until all particulates have settled to the bottom of the 200 mL beaker.
11. Filter the calcium oxalate solution through a Buchner funnel and two stacked 8 µm pore-size ashless Wattman 42 filter papers under vacuum. The filtrate should be collected in a round bottom flask and re-run through the filter paper until the filtrate is completely clear.
12. Allow the vacuum to continue to pull on the filter paper until dried. Once fully dried, remove the filter papers and place them in a crucible for calcination.
13. Proceed with the calcination process described in Steps 2–4 of Section 3.1.1.

## 4. Expected Results

As this protocol is both modular and large in scope, there are several points in the procedure where it is recommended to assess successful formation of the intermediate products between steps prior to continuing onto the next. Consequently, the measurable results for each portion of the procedure are presented below in association with their corresponding steps.

### 4.1. Synthesis of Injectable [^43^Sc]Sc-PSMA-617 from [^42^Ca]CaCO_3_

#### 4.1.1. Cyclotron Production of ^43^Sc

**Figure 3:**
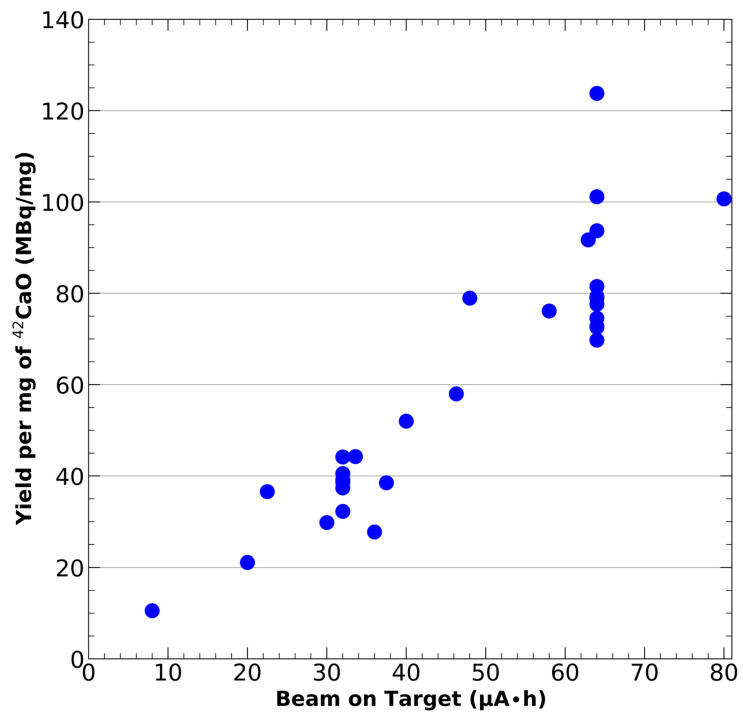
Target mass normalized yield of 43Sc as a function of beam current multiplied by beam time. Yields were obtained via the 42Ca(d,n)43Sc reaction using a biomedical cyclotron equipped with a 9 MeV deuteron beam (IBA, 18/9 MeV). Each datapoint represents an individual production of 43Sc.

At the time of this publication, no experimental data exists that describes the cross-section of the ^42^Ca(d,n)^42^Sc reaction or the stopping power of deuterons in CaO. It is possible to calculate an estimated yield of the ^42^Ca(d,n)^42^Sc reaction using simulation derived parameters for the cross-section and stopping power, but we have previously found these estimates to be inaccurate, as we have described in Meier et al. [50]. In the absence of a reliable, universal yield estimation metric for this procedure, in Figure 3 we have provided the end of bombardment yield (per mg of target of target material) as a function of the number of microamp-hours the target was exposed to for 30 separate ^43^Sc productions using a 9 MeV deuteron beam on an IBA cyclotron and a 7 mm diameter target. For these irradiations, the [^42^Ca]CaO target mass ranged from 4.6–22.4 mg (3.3–16.2 mg of ^42^Ca mass) with a mean target mass of 14.5 mg. Naturally, these results will vary from any experiment which utilizes a different target geometry, ^42^Ca purity, or beam energy.

**Figure 4:**
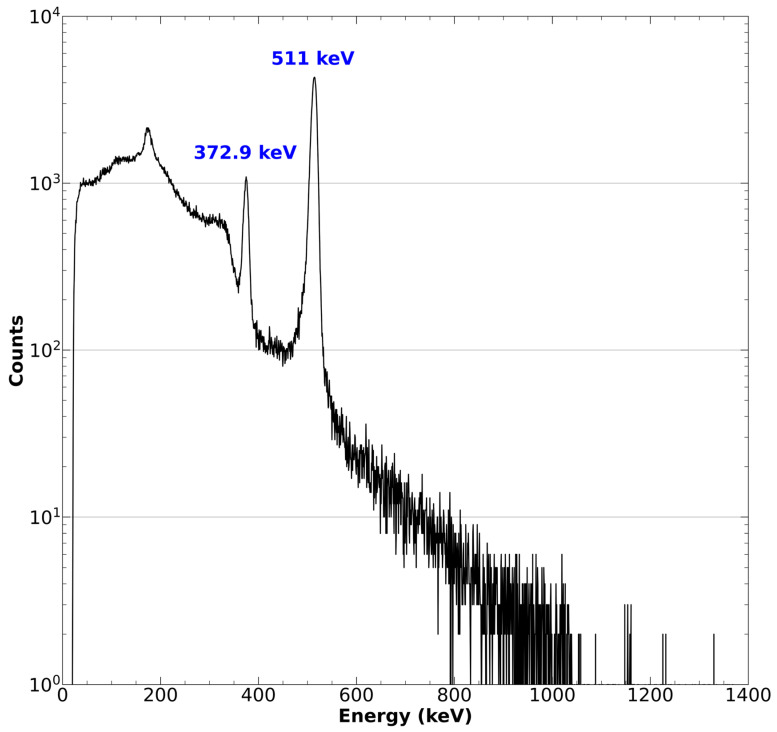
The gamma spectrum of 42Ca(d,n)43Sc produced 43Sc as obtained with a CZT detector. The characteristic γ-ray of 43Sc is clearly present at 372.9 keV. The positron annihilation peak is also emphasized at 511 keV. This figure was adopted from Meier et al. [50].

The isotopic identity of the produced radioactivity should also be verified utilizing HPGe, CZT, or NaI spectroscopy, especially when producing ^43^Sc for the first time or when utilizing a new or recycled source of target material. The resultant spectra should exhibit a prominent photopeak at 373 keV, which is clearly resolvable from the Compton continuum derived from the positron annihilation gamma rays. Naturally, the annihilation peak at 511 keV from ^43^Sc’s positron emission should also be clearly resolvable. Any other photopeaks within the spectra, aside from those derivatives of backscattering, should be investigated to ensure the purity of the radioactive product [41]. An example spectrum of cyclotron produced ^43^Sc obtained with a CZT detector has been adopted from Meier et al. and is shown in Figure 4 [50].

#### 4.1.2. Separation of ^43^Sc from [^42^Ca]CaO

The separation portion of the described protocol should terminate with the successful elution of [^43^Sc]ScCl_3_ from the DGA column, with minimal activity loss during the loading and washing of the DGA column. If a significant amount of activity (>10%) is lost in the loading process, it would suggest that the scandium is only weakly binding to the DGA which could indicate that the loading step was performed too rapidly. Similarly, if a significant amount of activity (>10%) becomes trapped in the DGA column, it suggests either that the elution was performed too rapidly, or that there is an issue with the HCl eluent, such as an incorrect concentration or insufficient temperature.

Table 1 is provided as a characterization of the activity loss expected at each step of the separation process decay, corrected to the start of the procedure and normalized by the starting activity. Each provided value is therefore the mean across 10 separate experiments, and errors are reported as the standard error. As is illustrated, no single step accounts for an average loss of more than 10.0% of the total activity, and any major deviation from that level of activity loss should be thoroughly investigated. The example mean activity fraction measured in each aliquot, as shown in Table 1, also provides a representative distribution of the elution of [^43^Sc]ScCl_3_ and can be used to infer which aliquots are the most likely to contain the highest concentration of activity.

#### 4.1.3. Synthesis of [^43^Sc]Sc-PSMA-617

**Figure 5:**
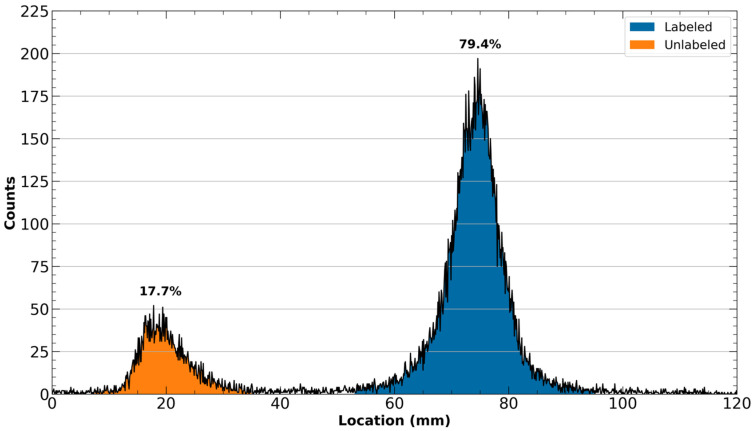
A representative radio-iTLC chromatogram exhibiting the unlabeled [^43^Sc]ScCl3 (left) and labeled [^43^Sc]Sc-PSMA-617 (right) peaks using 50:50 1 M ammonium acetate and methanol as the mobile phase following the radiolabeling of [^43^Sc]Sc-PSMA-617.

The resultant radio-TLC chromatogram obtained in Step 30 should exhibit two clearly separated peaks, one near the origin where the plate was spotted, and the other near the terminal location of the mobile phase. It is possible that only a singular one of these peaks may be observed if the labeling has completely succeeded (100% labeling) or failed (0% labeling). The first of these peaks represents unlabeled [^43^Sc]ScCl_3_, whereas the second peak is the desired product, [^43^Sc]Sc-PSMA-617. A representative radio-TLC chromatogram is exhibited in Figure 5.

The integrated number of counts within the [^43^Sc]Sc-PSMA-617 peak divided by the total number of counts throughout the entire chromatogram should indicate the overall percentage of radiolabeled [^43^Sc]Sc-PSMA-617 in the reaction mixture. For quality control purposes, the total percentage of radiolabeled [^43^Sc]Sc-PSMA-617 should be greater than 90%. Any radiolabeling yield below 90% is indicative of a target molar activity (chosen in Step 28) that was too high for the activity concentration of the reaction mixture or the potential presence of a metal contaminant.

As any unlabeled ^43^Sc will be removed in the purification portion of this protocol, it is still reasonable to proceed with poorer radiolabeling yields, but the excess unlabeled ligand will diminish the molar activity and thus the pharmacokinetics of the final compound.

#### 4.1.4. Purification and Quality Control of [^43^Sc]Sc-PSMA-617

**Figure 6:**
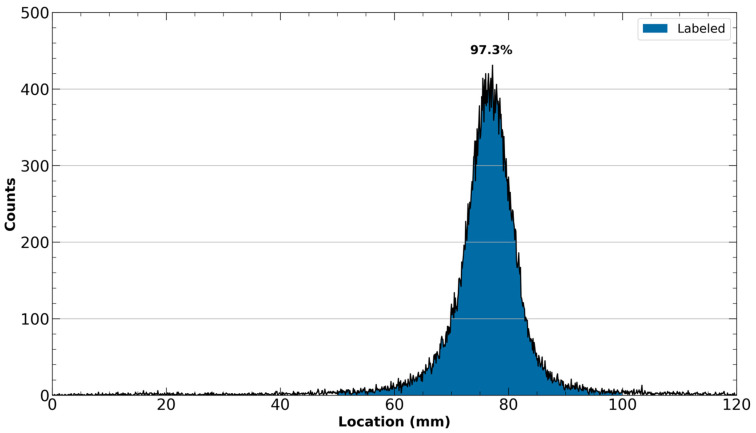
A representative radio-iTLC chromatogram exhibiting the sole labeled [^43^Sc]Sc-PSMA-617 peaks using 50:50 1 M ammonium acetate and methanol as the mobile phase following the purification of [^43^Sc]Sc-PSMA-617.

The loading of the Oasis cartridge should result in a pass-through solution containing the entirety of the unlabeled ^43^Sc activity with minimal amounts of activity also being removed in the water wash. As all the unlabeled activity has been removed, the radio-TLC collected from the eluate of the Oasis cartridge should exhibit only a single peak that moves with the solvent front, as is exhibited in Figure 6.

If there is not any planned in vivo usage of the synthesized [^43^Sc]Sc-PSMA-617, and thus reformulation is unnecessary, the true molar activity can also be determined by using a small sample of the final product (~25 μL) to generate a chromatogram with radio-HPLC. The retention time and the area under the peak from the UV trace of the generated chromatogram can then be compared to a standard curve generated from the UV trace of unlabeled PMSA-617, thus facilitating calculation of the molar activity.

#### 4.1.5. Reformulation of [^43^Sc]Sc-PSMA-617

**Figure 7:**
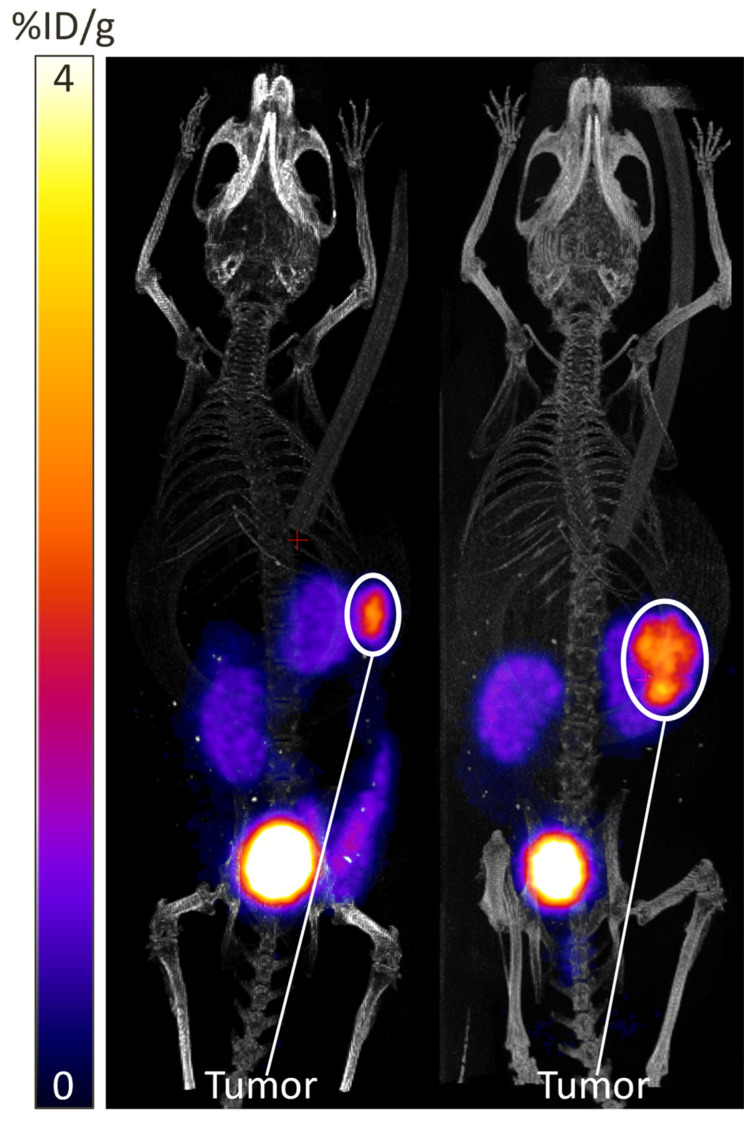
[^43^Sc]Sc-PSMA-617 PET images of two nude mice with enzalutamide resistant LNCaP xenografts at 240-min post-injection. The mouse in the left portion of the figure was injected with 5.21 MBq of [^43^Sc]Sc-PSMA-617 and the mouse in the right portion of the figure was injected with 5.15 MBq of [^43^Sc]Sc-PSMA-617. Both injections had a molar activity of 12.03 MBq/nmol of PSMA-617.

The reformulation of [^43^Sc]Sc-PSMA-617 into a PBS solvent enables the administration of [^43^Sc]Sc-PSMA-617 in vivo for the imaging of PSMA-expressing tissues. Under this lens, the successful synthesis and application of [^43^Sc]Sc-PSMA-617 in vivo should exhibit specific uptake in PSMA-expressing tumors, such as those derived from the LNCaP and 22Rv1 prostate cancer cell lines, as well as PSMA-mediated uptake in the salivary glands [57,58,59,60,61]. Non-specific uptake will also be observed in the kidneys and bladder, as PSMA-617-based radiopharmaceuticals wash out through the renal system [62]. PET images exhibiting the expected distribution of [^43^Sc]Sc-PSMA-617 in two athymic nude mice with enzalutamide resistant LNCaP (LNCaP-EnzaR) xenografts at 240-min post-injection are presented in Figure 6. Within Figure 7, a mouse with an LNCaP xenograft superimposed on top of the kidney from the axial view was deliberately chosen to exhibit tumor visualization near renal tissues at long time-points.

**Figure 8:**
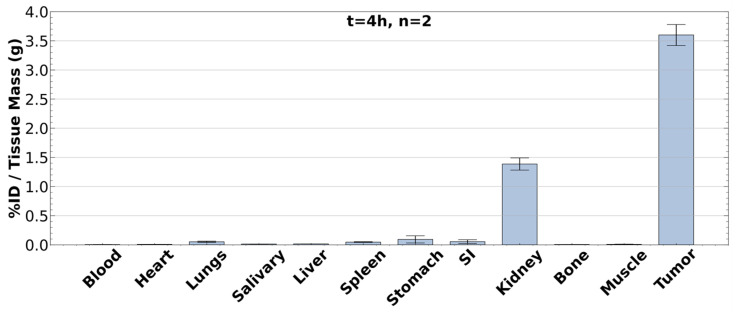
[^43^Sc]Sc-PSMA-617 ex vivo biodistribution of two nude mice with enzalutamide resistant LNCaP xenografts at 240-min post-injection. The values reported are the mean across both mice, and the presented error bars were calculated as the standard error.

Following the PET imaging, these two mice were also sacrificed and dissected for ex vivo biodistribution analysis. A transcardiac perfusion was performed to drain any radioactive blood components from the organs, and then samples of blood, heart, lungs, salivary glands, liver, spleen, stomach, small intestine, kidney, bone, muscle, and LNCaP tumor were harvested and massed. These samples were loaded into polystyrene tubes and placed into a Wizard2 2480 gamma counter, where the activity within each sample was measured. Normalizing the activity within each tissue sample by its mass produced the biodistribution presented in Figure 8.

As is shown, [^43^Sc]Sc-PSMA-617 should be clearly resolvable from all background tissues, including those of the renal system, by 240 min post-injection with minimal uptake in all non-renal background tissues. Although not exhibited here, PSMA-expressing tumors should be distinguishable from non-renal tissues by 20 min post-injection. Retention of [^43^Sc]Sc-PSMA-617 in PSMA-expressing lesions should be observed for at least 6 h beyond the initial injection, whilst non-specific uptake should decrease as the radiopharmaceutical is excreted through the renal system. Based on the literature conducted on [^177^Lu]Lu-PSMA-617, retention of the radioligand in PSMA-expressing tissues is expected well beyond the 6 h stated here; however, retention past this point has not been tested with [^43^Sc]Sc-PSMA-617 [63,64].

Some variation in the distribution of [^43^Sc]Sc-PSMA-617 in vivo should be expected due to variances in pharmacokinetics due to molar activity, PSMA expression in cell lines, and physiological differences between animals [65,66]. Conversely, if little to no uptake of [^43^Sc]Sc-PSMA-617 is observed in PSMA-expressing tissue, it is likely that the unlabeled PSMA-617 in the injected solution is in high enough concentration relative to [^43^Sc]Sc-PSMA-617 that it is acting as a competitive inhibitor and outcompeting [^43^Sc]Sc-PSMA-617 for PSMA-617 binding sites. This can be alleviated by improving the molar activity of the solution, with tumor localization capacity improving at higher molar activity.

### 4.2. Recycling of ^42^Ca Target Materials

The recovery rate of ^42^Ca from the waste materials collected in Section 3.1.4. is somewhat variable with our own experimental results, ranging from 65–90% recovery of the starting material. Possible explanations for this observed variability include loss of material in the transfer of recovery materials between containers and the inability to account for material loss in the target loading, target extraction, or irradiation processes. Therefore, it is recommended that recovery procedures be performed with multiple batches of irradiated targets to minimize the number of material transfers.

## 5. Reagents Setup

Prepare the following reagents prior to starting the protocol. Reagents that consist of the same core chemical (e.g., “HNO_3_”) are presented as a series of dilutions to minimize the handling of highly concentrated chemicals. For steps where a variable amount of reagent may be used, these preparations exhibit the precautionary case where excess reagent is needed.

### 5.1. Production of [^43^Sc]Sc-PSMA-617 Using Cyclotron Produced ^43^Sc

1. 5 M HNO_3_ (metal free)

**Reagent**

**Final Concentration**

**Amount**
Metal-Free HNO_3_ (15 M) 5 M5 mLTrace Analysis Water
10 mLTotal
15 mL

2. 2.5 M HNO_3_ (metal free)

**Reagent**

**Final Concentration**

**Amount**
Metal-Free HNO_3_ (5 M) 2.5 M3 mLTrace Analysis Water
3 mLTotal
6 mL

3. 1 M HCl (metal free)

**Reagent**

**Final Concentration**

**Amount**
Metal-Free HCl (12 M)1 M1 mLTrace Analysis Water
11 mLTotal
12 mL

4. 0.1 M HCl (metal free)

**Reagent**

**Final Concentration**

**Amount**
Metal-Free HCl (1 M)0.1 M1 mLTrace Analysis Water
9 mLTotal
10 mL

5. 1 M Ammonium Acetate Buffer (pH 5.25)

**Reagent**

**Final Concentration**

**Amount**
Ammonium Acetate~1 M0.78 gTrace Analysis Water
10 mLAcetic AcidVariable ^1^Variable ^1^Total
>10 mL^1^ Acetic acid is added to the aqueous ammonium acetate solution until the solution has reached pH 5.25. Consequently, the amount added and the concentration of acetic acid in the final reagent are not fixed quantities. As such, it is recommended that acetic acid be gradually added to the aqueous ammonium acetate in 100 μL increments and that the pH is checked with a benchtop pH meter/electrode after each increment until the desired pH is reached.

6. PSMA-617 (aqueous)

**Reagent**

**Final Concentration**

**Amount**
PSMA-6170.96 mM50 μgTrace Analysis Water
50 μLTotal
50 μL

### 5.2. Recycling of ^42^Ca Target Materials

1. 3 M HCl (metal free)

**Reagent**

**Final Concentration**

**Amount**
Metal-Free HCl (12 M) 3 M6 mLTrace Analysis Water
18 mLTotal
24 mL

2. 1 M HCl (metal free)

**Reagent**

**Final Concentration**

**Amount**
Metal-Free HCl (3 M)1 M4 mLTrace Analysis Water
8 mLTotal
12 mL

3. 0.3 M Ammonium Oxalate

**Reagent**

**Final Concentration**

**Amount**
Ammonium Oxalate 0.3 M0.45 gTrace Analysis Water
20 mLTotal
20 mL

4. 0.64 M Ammonium Hydroxide

**Reagent**

**Final Concentration**

**Amount**
Ammonium Hydroxide (5.65 M)0.64 M1.36 mLTrace Analysis Water
11.64 mLTotal
12 mL

## Figures and Tables

**Table 1:** The distribution of the mean fraction of decay-corrected activity observed in the elution of [^42^Ca]CaO and [^43^Sc]ScCl_3_ through a DGA column during the chemical separation phase of [^43^Sc]Sc-PSMA-617 production. Reported values are the mean across 10 experiments and the errors presented were calculated as the standard error across those 10 experiments. Bolded values indicate activity loss during the procedure.

Step	Description	Decay Corrected Activity (%)
20	Starting Activity	100
20	Remnant in PFA	**3.02 ± 0.769**
21	Pass-through solution	**0.134 ± 0.036**
21	Loaded DGA	92.0 ± 0.954
22	5 M HNO_3_ Wash	**0.051 ± 0.017**
23	1 M HCl Wash	**2.90 ± 0.594**
24	Elution Aliquot #1	0.246 ± 0.063
	Elution Aliquot #2	3.51 ± 1.80
	Elution Aliquot #3	10.4 ± 3.33
	Elution Aliquot #4	13.7 ± 1.71
	Elution Aliquot #5	17.0 ± 1.66
	Elution Aliquot #6	13.5 ± 1.50
	Elution Aliquot #7	10.5 ± 1.37
	Elution Aliquot #8	7.13 ± 1.17
	Elution Aliquot #9	4.44 ± 1.00
	Elution Aliquot #10	2.72 ± 0.726
25	Remnant in DGA	**3.80 ± 1.17**
**Total Activity Loss**		**9.91 ± 2.59**

## Data Availability

The data presented in this study are available on request from the corresponding authors. The data are not publicly accessible because they are not available in a format that is sufficiently accessible or reusable by other researchers.
